# The Effect of Number and Distribution of Mini Dental Implants on Overdenture Stability: An In Vitro Study

**DOI:** 10.3390/ma15092988

**Published:** 2022-04-20

**Authors:** Rafif Alshenaiber, Craig Barclay, Nick Silikas

**Affiliations:** 1 Division of Dentistry, Faculty of Biology, Medicine and Health, University of Manchester, Manchester M13 9PL, UK; nick.silikas@manchester.ac.uk; 2 Prosthetic Dental Sciences Department, College of Dentistry, Prince Sattam Bin Abdulaziz University, AlKharj 16278, Saudi Arabia; 3 Restorative Dentistry, University of Manchester Dental Hospital, Manchester M15 6FH, UK; craig.barclay@manchester.ac.uk

**Keywords:** mini dental implants, overdenture, dental prosthesis retention, masticatory force

## Abstract

The rotational movement of mini dental implants (MDIs) overdenture disturbs the function of the prosthesis. Many dentists place more MDIs to improve the overdenture stability; however, the influence of the MDIs number and distribution on the overdenture resistance to para-axial dislodgment has not been investigated. Seven resin models simulating atrophic mandibles housed twenty MDIs placed according to seven arrangements. Acrylic overdentures were fabricated for each cast and were dislodged five times in lateral, anterior and posterior directions, and the peak load dislodgment was measured. Each overdenture underwent 540 axial removal/placement cycles. The para-axial dislodgments were measured again, and data were compared. Dislodgment force values were measured in all directions, and the data were analysed using analysis of variance ANOVA and post hoc (*p* < 0.05). After six months of simulated placement/removal, increasing the MDI number showed a difference in resistance to para-axial dislodgment. The distribution affected the resistance to dislodgment in some directions. The inter-implant distance of 27 mm provided better resistance to posterior dislodgment than placing two MDIs close together at 19 mm. The placement of three MDIs at any distribution showed no significant difference except for resistance to posterior dislodgment. FourMDIs placed at any distribution showed a significant difference in all groups in all tested directions. The resistance to the para-axial dislodgment of MDI overdenture could improve with the increasing MDIs number and careful planning of MDI distribution.

## 1. Introduction

Prosthesis stability is an essential criterion that affects its success [1]. While removable prosthesis movement is expected during function, the resistance of lateral and anterior–posterior dislodgment forces describes the overdenture stability [2]. Once placed in the mouth to function, the overdenture is subjected to various forces in multiple directions [3]. Even the distribution of implant attachments within the arch helps in forces distribution, which reduces mechanical complications [4]. The overdenture is affected by pull-out forces during chewing, which leads to movements over the residual ridge. Stresses within the attachments can be created following prosthesis movement. Such stresses may result in plastic deformation of the attachment housing [5,6]. Consequently, attachment wear occurs, and then the progressive loss of retentive forces can lead to denture looseness or perhaps a fracture within the attachment system [7]. Using two implants to provide good overdenture stability for atrophic mandibles was suggested to reduce the cost of the treatment [8]. The placement of two MDIs was a successful treatment option compared to two conventional implants overdenture [9,10]. Prosthetic MDIs can improve overdenture retention and stability [10,11]. It is a cost-effective, minimally invasive treatment for cases with limited bone available [9]. MDI overdentures could rehabilitate atrophic mandibles without reconstructive surgeries; therefore, it is a suitable option to manage edentulous elderly [9,10,11,12]. In dentistry, stress distribution is widely evaluated using finite element analysis (FEA) [13]. It can be used to simulate the strain pattern and evaluate marginal bone loss around dental implants [14]. One study that utilised FEA found that more displacement of the overdenture was noticed with one and two MDIs compared to the same number of conventional implants [15]. Such a difference may be due to the size of the MDIs where the attachment has less surface area than conventional implants attachment. Therefore, increasing the MDI number might overcome the reduced size issue. The placement of more MDIs positively impacted the overdenture stability during function [16]. However, increasing the implant number is not always permitted and is case-dependent.

Determining the appropriate number of implants depends on many factors. It depends on the prosthesis type, which can be determined by the diagnostic findings and, most importantly, listening to the patient expectations [17]. Examination of the residual ridge, including soft tissue thickness and hard tissue quantity and quality, is essential in determining the implant number, length and angulation [18]. Mandibular removable prosthesis support is affected by the reduced area of the mandible and mobility of the floor of the mouth and the tongue [19,20]. Hence, one study suggested that using only two MDIs to support and retain overdenture was better accompanied by a sufficient residual ridge to share the load [16]. However, when more than two implants are planned for placement, the implant distribution is vital to permit restorability, aesthetics and maintenance. The test in this study was carried out to assess the MDI overdenture resistance to para-axial dislodgments with different MDI numbers and distribution by subjecting the MDI overdenture to dislodging rotational forces applied in a para-axial manner before and after six months of simulated placement/removal. The null hypothesis is that the number and distribution of MDIs supporting an overdenture placed in an atrophic mandible have no effect on their ability to resist para-axial and rotational movements.

## 2. Materials and Methods

**Figure 1:**
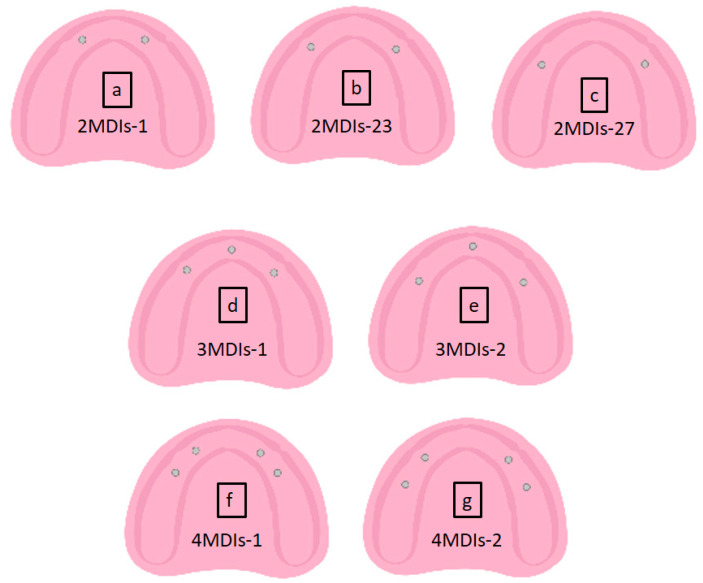
The MDIs were distributed according to their number into seven groups: for the two MDIs groups, (**a**) at lateral incisors area 19 mm apart (2MDIs-1), (**b**) at canine area 23 mm apart (2MDIs-23) and (**c**) at first premolar area 27 mm apart (2MDIs-27). In the three MDIs group, one MDI was placed at the midline and two MDIs were arranged (**d**) at the canine area (3MDIs-1) or (**e**) at the premolar area (3MDIs-2). The four MDIs group was distributed in 2 arrangements; (**f**) 2 posterior MDIs placed at the first premolars area and 2 anterior MDIs placed at the area of the lateral incisors (4MDIs-1), and (**g**) 2 posterior MDIs placed at the second premolars area and 2 anterior MDIs placed at the canines area (4MDIs-2).

Seven testing resin models simulating atrophic mandible with no undercuts were prepared using Orthoresin (Dentsply International, Inc., Milford, DE, USA). Twenty ILZ MDIs from Southern implants^®^ (Irene, South Africa) (2.4 mm × 13 mm) were placed according to seven arrangements using a dental surveyor (Austenal^®^ Surveyor, Skillbond Direct Ltd, Buckinghamshire, UK) to ensure parallel placement between MDIs within one cast (Figure 1).

Five acrylic overdenture replicas (Selectaplus—Dentsply International, Inc., Milford, DE, USA) were fabricated for each cast (n = 35), containing MDIs attachments standard micro insert from Rhain83 Company supplied with the MDIs.

Four cup hooks were incorporated into each acrylic overdenture: two were placed at each first molars area, and two were placed bilaterally at the canine region. At the top of the four chains, a pivoting joint was used to ensure even removal of overdenture [21]. A testing stand was connected to Chatillon^®^ digital force series II (Ametek GB Ltd ta Lloyd Instruments, West Sussex, UK) remote non-dedicated gauge (DFS II-R-ND) to measure the peak load (N) required for attachment dislodgment.

The peak load or attachment break was graphically recorded and analysed using the Nexygen DF2 software. The chains were adjusted to prevent slacking before each testing cycle, and the force gauge was set to zero.

**Figure 2:**
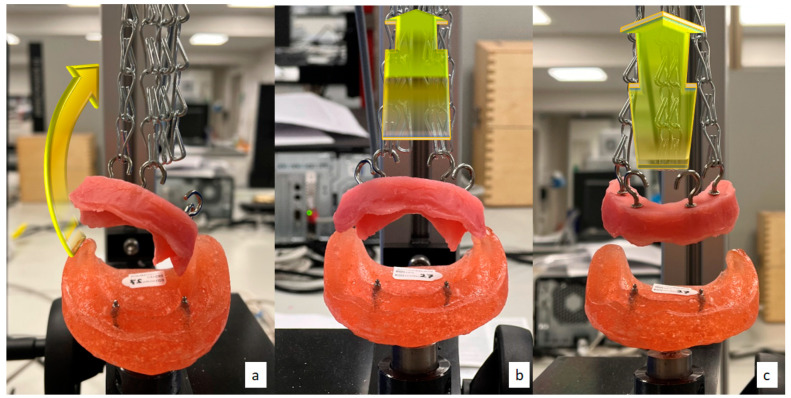
Three oblique dislodgments: (**a**) right lateral, (**b**) anterior and (**c**) posterior directions.

Three types of two-point oblique dislodgment were carried out:

1. Lateral (oblique) dislodgment: connecting two chains on the hooks at the right side and disconnecting the left side chains (Figure 2a). In addition, the left side dislodgment test was also carried out, but values were excluded to reduce data analysis.
2. Anterior rotational dislodgment: connecting two chains on the hooks placed anteriorly and disconnecting the posterior side chains (Figure 2b).
3. Posterior rotational dislodgment: connecting two chains on the hooks at the posterior at both sides and disconnecting the anterior chains (Figure 2c).

All four chains were connected after recording the initial para-axial dislodgments in all directions, and 540 consecutive removal/placement cycles were carried out to simulate six months of placement and removal. We cannot determine how many cycles of oblique dislodgments would simulate in vitro function for this test. Therefore, after completing the testing cycles, lateral and rotational dislodgment forces were measured again similarly to pre-testing and data were compared between groups.

The force dislodgment measurements were recorded in each direction: pre-load values and post-load values following the completion of 6 months of simulated placement/removal cycles. The means of the dislodgment forces were recorded, and the data were analysed using SPSS Version 25.0 (IBM Corp. Released 2016. IBM SPSS Statistics for Windows, Version 25.0, Armonk, NY, USA: IBM Corp.) using one way ANOVA (*p* < 0.05). In the event of significant differences, Tukey’s post hoc multiple comparison tests were utilised to determine the difference (*p* < 0.05).

## 3. Results and Discussion

Many studies reported that overdenture stability improves the patient satisfaction with overdenture [22]. Patients reported that the rotation of overdenture interfered with their mastication [23]. Therefore, assessing overdenture resistance to para-axial dislodgment is essential for its clinical success. However, the complexity of the overdenture movements in the oral cavity limit the in vitro simulation of the testing environment to assess prosthesis stability. A directional dislodgment test was used in several studies to determine the retention and stability of the prosthesis [24,25,26,27,28,29,30]. Unfortunately, in a laboratory setting, we cannot estimate the amount or frequency of the para-axial dislodgment forces. We know such forces occur during function; therefore, several attempts were used to enhance the removable prosthesis stability by resisting such forces. Many suggested increasing the implant number to improve the overdenture stability [16,29,31]. Consequently, the results of this study agree with the literature, as increasing the MDIs number showed an increased resistance to lateral and anterior-posterior rotational forces. Regarding the distribution of implants within the arch, the presence of the anterior implant provided indirect retention during the posterior dislodgment of overdenture, increasing the stability [32].

### 3.1. Initial Dislodgment Forces

The mean initial dislodgment force values of the five samples in each group were measured in each direction. One way ANOVA showed a significant difference between the initial dislodgment measurements in each direction in all tested groups (Table 1).

Tukey post hoc was carried out to assess the level of significance between groups. All groups showed a significant difference in the dislodgment force in all directions. However, Table 2 shows no significant difference in the lateral and anterior dislodgment force between the two MDIs at all inter-implant distances.

No significant difference was found in the lateral dislodgment force between the three MDI groups regardless of their distribution, with *p* = 0.99. This similarly applied to the four MDIs groups, with *p* = 0.98.

A significant difference was found in the anterior rotational dislodgment force between the three MDI groups regardless of their distribution, with *p* < 0.00. This similarly applied to the four MDIs groups, with *p* = 0.01.

No significant difference was found in the posterior dislodgment force when two MDIs were placed at 19 mm and 23 mm, with *p* = 0.26. However, a significant difference was found when two MDIs were placed at 27 mm compared to 19 mm, with *p* < 0.00, and 23 mm, with *p* < 0.00.

In addition, no significant difference was found in the posterior rotational dislodgment force between the three MDI groups regardless of their distribution, with *p* = 0.07. In addition, no significant difference was found in the posterior rotational dislodgment force between the three MDI-2 and the four MDI-1, with *p* = 0.25. However, there was a significant difference between the four MDI-2 with the three MDI-1 and three MDI-2 at *p* < 0.00 and *p* < 0.00, respectively. In four MDI groups, no significant difference was found in the posterior rotational dislodgment force, regardless of their distribution (*p* = 0.21).

### 3.2. Dislodgment Forces Following Six Months of Simulated Placement/Removal

After completing the axial pulling cycles, the measurements were re-recorded for each sample, and mean dislodgment force values were measured in the same manner. All of the tested groups showed a significant difference in post-loading dislodgment measurements in all directions (Table 3).

Tukey post hoc was carried out to assess the level of significance between groups. For the lateral oblique dislodgment, all groups showed significant differences in the dislodgment force. However, no significant difference was found in the lateral and anterior dislodgment force between the two MDIs at all inter-implant distances (Table 4).

For the lateral oblique dislodgment, there was no significant difference in the three MDIs groups regardless of their distribution, with *p* = 1.00, or in the four MDIs placed in different distribution, with *p* = 1.00.

For the three MDIs group, there was no significant difference in both distributions, with *p* = 0.08 at the anterior dislodgment. However, there was a significant difference between three MDIs and four MDIs at all distributions, with *p* < 0.05. For the four MDIs groups, there was a significant difference in the anterior dislodgment for all other MDI numbers and distributions. Even between the four MDIs at all distribution, there was a significant difference, with *p* = 0.00.

There was no significant difference in the posterior rotational dislodgment when two MDIs were placed at a 19 mm or 23 mm inter-implant distance, with *p* = 0.98, or at 23 mm and 27 mm, with *p* = 0.18. However, there was a significant difference between the 19 mm and 27 mm inter-implant distance, with *p* = 0.036. The presence of significance can be due to the larger difference between the 19 mm and 27 mm.

The number and distribution of MDIs showed a difference in dislodgment measurements in all directions. One study revealed that the retention force of stud attachments differs according to the direction of dislodgment [30]. The mechanical advantage is the ratio of the length of the power arm to the length of the resistance arm. Understanding the biomechanics in overdenture movement during function involves understanding lever movements [33]. All three orders may occur with dislodging forces causing anteroposterior overdenture movements.

**Figure 3:**
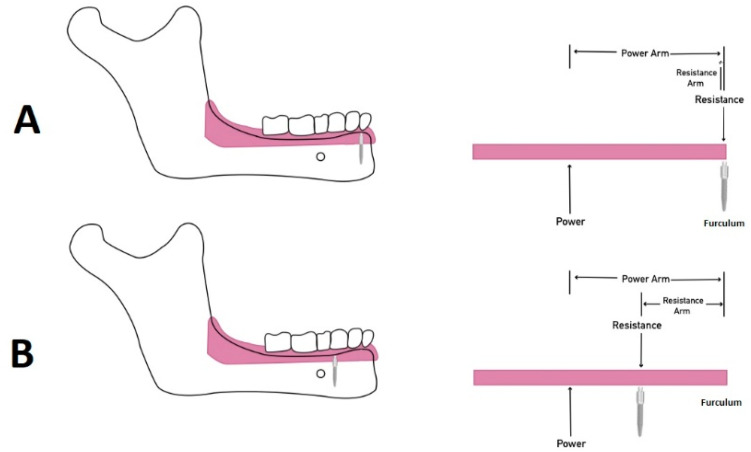
Class I and II lever systems with anterior residual ridge act as a fulcrum, MDI serves as resistance and power is the posterior dislodging forces: (**A**) Class I lever system showing that a short resistance arm as the fulcrum and resistance are coincident. (**B**) Class II lever system occurs by moving the MDI posteriorly, which lengthens the resistance arm, improving mechanical advantage.

The biomechanical lever movements agree with the results of this study. Implants to support overdenture can act as a class I lever movement fulcrum. The posterior extension acts as the lever arm, and the distance to the anterior teeth acts as the other lever arm. The force applied at the posterior or anterior lever arms produces posterior or anterior rotation. When the implant (furculum) is placed far from the loading point (posterior area during chewing), more denture rotation or dislodgment will occur, even with minimal loading. Several studies showed that an increased inter-implant distance increased the dislodgment forces [34,35,36]. Therefore, when only two implants are placed at the anterior mandible, we suggest placing them as far apart as possible by increasing the inter-implant distance to 27 mm to better resist posterior rotational forces (Figure 3).

**Figure 4:**
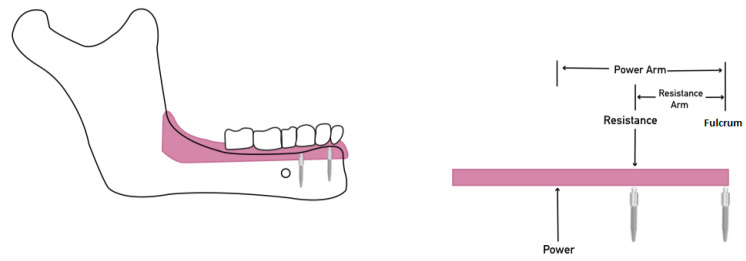
Class II lever system with anterior MDI act as a fulcrum, posterior MDI act as a resistance and the power is the posterior dislodging force. Placement of posterior MDI create a resistance point; notice the long resistance arm resulting from widely placing MDIs improving the mechanical advantage.

Placing another MDI between the furculum MDI and posterior extension produces a class II lever, where the resistance point (second MDI) prevents posterior rotation (Figure 4). Hence, increasing the number of MDIs provided more resistance to posterior movements [16].

For the three MDIs, regardless of the distribution, there was a significant difference in the posterior dislodgment force with all tested groups. The *p* value of 0.001 showed a difference between three MDI at both distributions. Similarly, the four MDIs groups significantly differed in the posterior dislodgment force with all tested groups. Additionally, there was a significant difference between both distributions of four MDIs, with *p* = 0.019.

Closely distributed MDIs showed reduced resistance to posterior dislodgment. The class II lever occurs when the MDIs are close to the midline, where dislodgment of the posterior MDIs attachments results in a pivot point where overdenture rotates. Similarly, a finite element analysis showed that the placement of a midline implant between two implants resulted in rotation of the overdenture [14].

**Figure 5:**
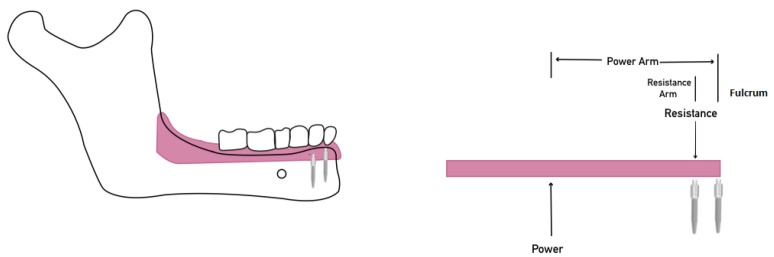
Class II lever system with anterior MDI act as a fulcrum, posterior MDI act as a resistance and the power is the posterior dislodging force. Due to the close placement between the anterior and posterior MDIs, the short resistance arm reduces the mechanical advantage.

Therefore, the posterior MDIs proximity to the midline reduces the overdenture rotation around the fulcrum. Such a reduction in resistance to dislodgment can be related to the prosthesis (Figure 5). In the same way, the overdenture anterior–posterior stability improved as the implant moved posteriorly [34]. 

The atrophic mandible resorption patterns cause a lingual shift in the teeth position over the residual ridge [18,37]. This may affect the lower face profile, requiring teeth setting labially over the residual ridge. However, such a teeth position will increase the risk of anterior overdenture rotation [23]. Therefore, in this case, we suggest increasing the MDIs number and broadly distributing them within the arch to reduce rotation and increase stability.

**Figure 6:**
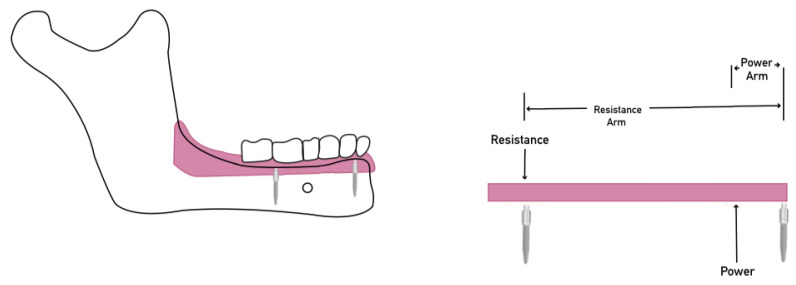
Class III lever system with anterior MDI act as a fulcrum, posterior MDI act as a resistance and the power is the anterior dislodging force. Placement of posterior MDI create a resistance point toward the anterior dislodgment force; notice the long resistance arm resulting from placing MDI posterior to the dislodgment force.

The placement posterior MDIs as possible posteriorly creates lever-III-to-anterior dislodgment forces. The resistance to the dislodgment of the posterior MDIs prevents the anterior overdenture rotation (Figure 6). In this study, we found that the further placement of the posterior MDI resulted in a better resistance to anterior dislodgment when the same number of MDIs were placed. 

**Figure 7:**
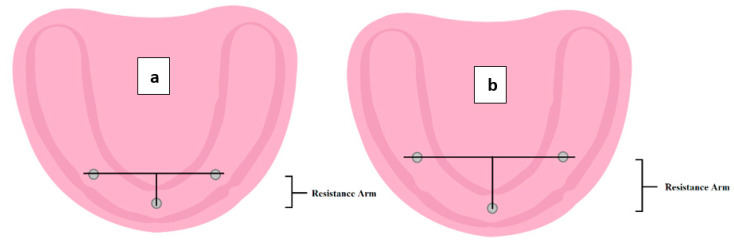
The difference in resistance arm length; note the increase in the length of the resistance arm in model (**b**) compared to (**a**) by moving the two posterior MDIs distally.

The length of the resistance arm is between the midline MDI (near fulcrum) and the line bisecting the posterior MDIs. The closer the posterior MDIs to the midline, the shorter the resistance arm and the faster the rotation. Increasing the length of the resistance arm by moving the two posterior MDIs distally results in a greater increase in overdenture resistance to dislodgment, as more power would be required to remove the overdenture. (Figure 7).

This investigation showed that resistance to dislodgments varied according to the direction of the dislodgment. Both initial and post-load values were consistent among the tested groups, agreeing with a similar study. Maxillary overdenture retained by four MDIs with O-rings recorded more resistance towards posterior dislodgment, followed by lateral and anterior forces [38]. In the same way, forces that resisted posterior dislodgment in this study were higher than those that resisted anterior or lateral dislodgment for the three and four MDIs groups. Another in vitro study reported similar results, as the overdenture posterior dislodging forces were higher than those required to dislodge the overdenture vertically and laterally [32]. However, the resistance to anterior dislodgment forces of the two MDIs groups was higher than the posterior and lateral resistance. As mentioned earlier, such a result may be expected with the attachments placed anteriorly.

It was observed that the placement of three MDIs with the two posterior MDIs at the first premolar showed a similar resistance to posterior dislodgment with the four MDIs group when the two posterior MDIs were also placed at the first premolar. Therefore, when anatomical limitations govern the placement of MDIs posteriorly (beyond canine or at premolar area), we suggest placing three widely distributed MDIs rather than four close MDIs to reduce the treatment cost. Anatomic limitations, such as mental foramen, accessory inferior alveolar nerves or even extreme posterior bone loss, can also favour this option. The observed results of this test can be used as a helping guide when planning MDIs-retained overdenture. Patients struggling with overdenture stability might benefit from adding more than two MDIs based on this study. Moreover, lateral dislodgment forces that occur during mastication may be more pronounced when patients chew on one side [39]. Therefore, patients who habitually chew on one side or struggle with skeletal or muscular dysfunctions can benefit from adding more MDIs.

This study has several limitations, as replicating clinical conditions during testing is more complex than one laboratory test. The in vitro testing used did not consider many aspects, such as the residual ridge anatomy, presence of soft tissue and forces applied to overdenture. Therefore, developing a testing protocol for dental attachments is needed for further studies on each factor using well-controlled standards. The only testing protocol for dental attachment is the ISO 13017 specified to test magnet attachment properties [40]. Thus, the model design and testing methodology were based on reported studies that utilised the same objectives [29,34]. 

One factor that was discounted in the model design is the effect of the bearing area on the denture resistance to para-axial dislodgments [41]. Thus far, the research aims to study the overdenture stability based on the MDIs ability to resist para-axial dislodgment, not the effect of tissue support. The solo resin cast design was used to avoid the influence of cofounders’ variables on the measured values. In addition, soft tissue replacement was ruled out because it interfered with attachment dislodgment force measurement. Moreover, the testing environment lacks an opposing arch, which clinically plays a role in denture stability [42].

Saliva, the cheeks, lips and tongue contribute to the force application and overdenture stability; all those factors were excluded from the scope of this study. Not using any lubricant medium may result in excessive friction during the dislodgment of attachments [43]. However, several factors justify not using saliva replacement, including the difficulty in testing all samples using the same amount of saliva and maintaining the saliva flow and temperature within the testing period. Moreover, some studies suggested that attachments could absorb water, causing a transient increase in the dislodgment force [44,45]. Further studies to include different attachment types, as they can exhibit different behaviour toward resisting dislodgment forces, are recommended [30,34,46]. Based on the limitations of this in vitro study, the results should be considered cautiously, and further experimental and prospective clinical studies should be carried out to validate the results.

## 4. Conclusions

Within the limitation of this in vitro study, the resistance to lateral and anterior-posterior dislodgement of MDIs-retained overdenture could improve with an increasing MDIs number and careful planning of the MDI distribution within the atrophic mandibular. The MDIs distribution affected the resistance to anterior-posterior dislodgement but not the resistance to lateral dislodgement. Two MDIs at an increased inter-implant distance of 27 mm improved the resistance to posterior dislodgements compared to closely placed MDIs. When a third MDI is considered in the midline, the placement of posterior MDIs further away would also improve the resistance to posterior dislodgement. Similarly, a wide distribution by increasing the distance between the anterior and posterior four MDIs improved the overdenture resistance to anterior–posterior dislodgment. Nevertheless, widely distributed three MDIs rather than closely distributed four MDIs provide a similar posterior dislodgment resistance while reducing the treatment cost.

## Figures and Tables

**Table 1:** The initial mean ± standard deviation of force to dislodgement in three directions, * *p* < 0.05.

	Force to Dislodgment (N) ± SD							One Way ANOVA *p* Value
Dislodgment Force Direction	2MDIs-19	2MDIs-23	2MDIs-27	3MDIs-1	3MDIs-2	4MDIs-1	4MDIs-2	
Right lateral oblique	22.20 ± 0.30	22.84 ± 0.76	22.20 ± 0.61	27.83 ± 0.49	27.99 ± 0.62	34.72 ± 0.28	34.98 ± 0.28	0.00 *
Anterior rotational	27.91 ± 0.68	28.16 ± 0.43	28.20 ± 0.68	32.02 ± 0.45	34.81 ± 0.35	38.04 ± 0.16	39.17 ± 0.27	0.00 *
Posterior rotational	25.92 ± 0.57	26.72 ± 0.65	28.79 ± 0.68	36.99 ± 0.49	38.03 ± 0.31	38.84 ± 0.63	39.69 ± 0.33	0.00 *

**Table 2:** The significance level of Tukey post hoc test for lateral and anterior dislodgment within two MDIs groups; the mean difference is significant at the 0.05 level.

The Inter-Implant Distances Groups	*p* Value at Lateral Dislodgment	*p* Value at Anterior Dislodgment
19 mm vs. 23 mm	0.44	0.98
19 mm vs. 27 mm	1.00	0.95
23 mm vs. 27 mm	0.45	1.00

**Table 3:** The post-placement/removal mean ± standard deviation of force to dislodgement in three directions, * *p* < 0.05.

	Force to Dislodgment (N) ± SD							One Way ANOVA *p* Value
Dislodgment Force Direction	2MDIs-19	2MDIs-23	2MDIs-27	3MDIs-1	3MDIs-2	4MDIs-1	4MDIs-2	
Right lateral oblique	17.87 ± 0.31	18.42 ± 0.34	17.92 ± 0.66	22.70 ± 0.55	22.76 ± 0.39	28.43 ± 0.35	28.44 ± 0.58	0.00 *
Anterior rotational	28.11 ± 0.49	27.82 ± 0.33	27.72 ± 0.39	29.54 ± 0.33	30.24 ± 0.45	34.70 ± 0.25	36.70 ± 0.34	0.00 *
Posterior rotational	22.56 ± 0.31	22.87 ± 0.18	23.90 ± 0.31	31.65 ± 1.36	33.66 ± 0.27	35.85 ± 0.27	37.29 ± 0.79	0.00 *

**Table 4:** The significance level of Tukey post hoc test for post-placement/removal lateral and anterior dislodgments within two MDIs groups; the mean difference is significant at the 0.05 level.

The Inter-Implant Distances Groups	*p* Value at Lateral Dislodgment	*p* Value at Anterior Dislodgment
19 mm vs. 23 mm	0.53	0.88
19 mm vs. 27 mm	1.00	0.67
23 mm vs. 27 mm	0.63	1.00

## Data Availability

Data are available upon request from the corresponding author.
